# Risk of tuberculosis cattle herd breakdowns in Ireland: effects of badger culling effort, density and historic large-scale interventions

**DOI:** 10.1186/s13567-014-0109-4

**Published:** 2014-10-26

**Authors:** Andrew W Byrne, Paul W White, Guy McGrath, James O’Keeffe, S Wayne Martin

**Affiliations:** Centre for Veterinary Epidemiology and Risk Analysis, School of Veterinary Medicine, University College Dublin, Belfield, Dublin 4, Ireland; Current address: Veterinary Sciences Division, Bacteriology Branch, Agri-Food and Biosciences Institute, Stormont, Stoney Road, Belfast, BT4 3SD UK; Department of Agriculture, Food, and the Marine, Agriculture House, Dublin 2, Ireland; Department of Population Medicine, University of Guelph, Guelph, Canada

## Abstract

Bovine tuberculosis (bTB) continues to be a problem in cattle herds in Ireland and Britain. It has been suggested that failure to eradicate this disease is related to the presence of a wildlife reservoir (the badger)*.* A large-scale project was undertaken in the Republic of Ireland during 1997–2002 to assess whether badger removal could contribute to reducing risk of cattle herd breakdowns in four areas. During the period of that “four area” study, there was a significant decrease in risk in intensively culled (removal) areas relative to reference areas. In the present study, we revisit these areas to assess if there were any residual area effects of this former intervention a decade on (2007–2012). Over the study period there was an overall declining trend in bTB breakdown risk to cattle herds. Cattle herds within former removal areas experienced significantly reduced risk of breakdown relative to herds within former reference areas or herds within non-treatment areas (OR: 0.53; *P* < 0.001). Increased herd breakdown risk was associated with increasing herd size (OR: 1.92-2.03; *P* < 0.001) and herd bTB history (OR: 2.25-2.40; *P* < 0.001). There was increased risk of herd breakdowns in areas with higher badger densities, but this association was only significant early in the study (PD*YEAR interaction; *P* < 0.001). Badgers were culled in areas with higher cattle bTB risk (targeted culling). Risk tended to decline with cumulative culling effort only in three counties, but increased in the fourth (Donegal). Culling badgers is not seen as a viable long-term strategy. However, mixed policy options with biosecurity and badger vaccination, may help in managing cattle breakdown risk.

**Electronic supplementary material:**

The online version of this article (doi:10.1186/s13567-014-0109-4) contains supplementary material, which is available to authorized users.

## Introduction

Bovine tuberculosis (bTB), caused by *Mycobacterium bovis* infection, is a serious and protracted problem for cattle industries in the Republic of Ireland (ROI) and the United Kingdom (UK), where intensive intervention programs have failed to eradicate the disease [1,2]. Numerous studies have been undertaken to assess risk factors associated with cattle herd breakdowns (e.g. [3-8]), as a means of guiding policy formulation and assessing where interventions may yield the best outcomes in progressing toward bTB eradication [1,2,9,10]. The presence of wildlife reservoirs (predominantly the badger, *Meles meles*) have been highlighted as a major impediment to eradication of *M. bovis* infection in cattle in ROI and the UK [11,12]. Wildlife intervention strategies that reduce the risk of reinfection of cattle herds from wildlife include culling and bTB vaccination of badgers. Animal husbandry and biosecurity measures [2,12,13] can also reduce the risk of new infections. Different culling strategies have been implemented as part of scientific trials or government policies: proactive culling (large scale area-wide removal, repeated culling), reactive culling (local scale removal, single pulse cull in reaction to a cattle herd breakdown) and targeted culling (local scale removal, repeated culling in reaction to an initial cattle herd breakdown). In the Republic of Ireland, the current national strategy is the targeted (culling) removal of badgers in areas where large cattle herd breakdowns are identified (> 2 standard interpretation skin test reactors using the Single Intradermal Comparitive Tuberculin Test (SICTT)), where a local investigation found the presence of badgers being a potential risk factor [2,12,13]. Once badger setts are “recruited” into the culling regime, attempts to remove badgers at active setts are repeated on an annual basis (see [14,15] for greater detail).

During 1997 to 2002, four large-scale (~200 km^2^ each) badger removal programs (proactive culling) were undertaken as part of a culling trial within the Republic of Ireland [3]. During this period, badger population densities were reduced to as low levels as was possible using a trap and dispatch regime [3,16]. Each removal site was paired with a reference site, where limited removals took place (but where removals were not stopped) during the period of the study (see [3] for details). The effect of badger removal during the trial period was highly significant with reduced risk to herds within removal areas in comparison with reference areas [3]. Previous investigations have suggested that the beneficial effects of culling may continue for some time after the end of these culling trials (P. White, unpublished; S.W. Martin, unpublished). Furthermore, there is evidence of a similar beneficial effect persisting subsequent to badger removals in studies in Britain [17,18]. In the intervening period (2004–2012), across all areas of the four counties a targeted culling programme was introduced [2,12,13]. This situation allowed us to test the hypothesis that historic intensive culling could still be benefitting farms located in former removal areas relative to historic reference (comparative) areas. The relationship between the historic culls and recent breakdown risk would be, however, modulated by the underlying variation in density of badgers across landscapes [19] and also the targeted culling being undertaken. Therefore, we investigated if there were any benefits to farms, in terms of bTB breakdown risk, within removal areas a decade after the cull trial began (2007–2012) in comparison with reference areas using a similar approach to the original analysis [3]. We then extended the analysis to include the potential effects of heterogeneity in badger abundance (metrics of badger social-group density; [19]) and culling effort. This analysis is the first to bring together metrics of historic disturbance, underlying potential density (“carrying capacity” of the landscape) and metrics of removal intensity of a wildlife host, and model these effects on cattle herd bTB breakdown risk in Ireland.

## Materials and methods

### Badger capture

During the four area project period (1997–2002) and the present study period (2007–2012) badgers were captured using wire stopped restraints and dispatched by 0.22 calibre rifle. Wire restraints were placed in the proximity of active badger setts and along runs, in an attempt to increase the probability of capture [14]. Wire stopped restraints conform to national legislation for the capture of wildlife (Wildlife Act, 1976, Regulations 2003 (S.l. 620 of 2003) and result in very low occurrences of trap-related injuries [20,21]. All licensing, capturing and culling adhere to the standards specified in the Irish Wildlife Acts (1976 to 2010–section 23(6)(A)).

### Effect of historic proactive cull - Standard analysis

We implemented a comparable analytical approach to Griffin et al. [3], to create a baseline model for the effect of historic culls on breakdown herd risk. For convenience, we use the term “treatment” to represent an area of land that was associated with the former large-scale badger cull trial (four-area project; [3]; see Additional file 1). Within the treatment areas, reference areas had limited reactive culling undertaken during 1997–2002; removal areas underwent proactive area-wide intensive culling during 1997–2002 (see [3] for details; see Additional file 1). While culling efficacy was not explicitly estimated during the culling period, the current removal estimate for the period was > 85% in proactive areas [16], which is in keeping with estimates for the cumulative effects of repeated badger trapping found in other studies [14,22-24]. Our null hypothesis was that there was no significant difference between former treatment areas in terms of herd breakdown risk.

Like Griffin et al. [3], we modelled cattle bTB risk using a multivariable logistic regression, fitted using a Generalised Estimating Equations (GEE) framework to account for repeated measures on herds. We used a first order auto-regressive correlation structure. The outcome variable for this model was whether a cattle herd experienced a tuberculosis breakdown within a calendar year. Descriptive statistics for the study cohort is given in Tables 1 and 2. We defined a cattle herd breakdown, as one with two or more standard reactors from any test during the calendar year (all herds are tested annually using the Single Intradermal Comparative Tuberculin Test (SICTT); see [25] for details of the testing regime implemented in Ireland). Herds with visible lesions identified at slaughter, but where subsequent skin test positives were not identified were not included in this study (in contrast to [3]). This was due to known variation in the effectiveness of bTB factory surveillance [26,27]. Despite this, there was a strong correlation between standard reactors and lesion reactors. Furthermore, any animals that were found to be tuberculosis positive at slaughter (lesions detected) instigated a re-test of the herds. If two or more standard reactors were confirmed at re-test, the herd was considered to be a breakdown herd for that year.

**Table 1:** **Numbers of herds in each area and county of the study**

	**Reference**	**Removal**	**Non-treatment**	**Total**
**Cork**	236	264	11 017	11 517
**Donegal**	228	268	4810	5306
**Kilkenny**	223	238	2357	2818
**Monaghan**	518	733	2354	3605
**Total**	1205	1503	20 538	23 246

Reference areas were areas with limited culling during the former intervention study (four area study; 1997–2002); removal areas experienced intensive, repeated proactive culling during the four area study; Non-treatment areas were farms wholly outside of any former study areas, but with registered land within the county. Herd locations were taken from the Land Parcel (LPIS) dataset. Note: Not all herds entered every model, as some herds did not have full testing records for the year of the study (e.g. not farming for full duration of the study).

**Table 2:** **Numbers of observations (yearly test status for each registered herd) in each area and county of the study**

	**Reference**	**Removal**	**Non-treatment**	**Total**
**Cork**	1361	1516	62 187	65 064
**Donegal**	1252	1510	26 363	29 125
**Kilkenny**	1198	1286	13 314	15 798
**Monaghan**	2934	4182	13 415	20 531
**Total**	6745	8494	115 279	130 518

We also included mean herd size as a potential predictor. This was generated using the test dataset, averaging the number of cattle tested during a full herd test. Where herds did not have a full herd test during a calendar year (if they were follow-up or partial herd tests undertaken, for example), previous or subsequent full test herd size was used. We transformed herd size by taking its logarithm, as the distribution was highly skewed. Herds with a herd history of only one year without full herd test data were discarded from further analysis (*n* = 50 herds). Other predictors included calendar year, treatment site (a binary variable representing removal area or reference area) and county. Following Griffin et al. [3], we included a dummy variable (PH) for previous herd history (1 = herd had a previous breakdown; 0 = did not have a breakdown). To inform year 2007, we gathered yearly breakdown data for each herd from 2005 and 2006. For a herd to be included as being within a former exposed area, > 70% of the farm’s land had to be within a former treatment area.

### Extended analysis – metrics of badger density and culling effort

Similar to the previous approach (see above), we ran GEE models (logit link; binomial family; auto-regressive 1 correlation structure) with the outcome being whether a cattle herd had a breakdown during a year. In addition to the previous analysis, we extended our study population to include herds wholly outside of former project areas (Four area project) in counties Monaghan, Kilkenny, Donegal and Cork (non-treatment areas). We again controlled for the effects of YEAR (2007–2012; categorical fixed effects), PREVIOUS herd history (whether or not there was a herd breakdown), HERD SIZE (log transformed) and COUNTY (categorical). We used cattle herd data from 2005 and 2006 to inform previous history for 2007.

We were particularly interested in the effects of badgers on herd breakdown risk. We added a variable which represented a metric of badger social group density (derived from the maps within [19]), as a means of controlling for underlying heterogeneity of badger density. This variable represents the suitability of landscapes for social group density based on main sett abundance [28,29]. This metric was scaled to values between zero and one; higher values denote higher densities (see [19] for details). We denote this variable POTENTIAL DENSITY, as the variable is invariant to culling effort (main setts will exist after a culling operation), and could be considered a metric of the carrying capacity of the landscape for badgers. The predictions from the biogeographic model (1 ha scale) were averaged for the area of each farm, such that each farm had one suitability value.

We derived a spatially explicit metric of badger culling effort over time (CULL). Badgers captured were summed by sett for each year, 2004 through to 2013. A point density analysis (ArcGIS 10.1, ESRI, Redlands, CA, USA) was performed on sett location for each year with number of badgers captured per sett as the population field. A circular neighbourhood with a 500 metre-radius was chosen with an output grid of 100 × 100 metres. The radius of 500 m was selected to represent an approximate mean halfway distance between main setts in the Republic of Ireland [14]. Where buffers overlapped, badgers were counted cumulatively, thus this metric is a relative measure of culling intensity. The unit area of the density analyses performed was square kilometres. To correct the density value to actual number of badgers per square kilometre per 100 × 100 metre grid-square, the raster value was multiplied by the area of the 500 metre circle (0.785 square kilometres). A point density analysis was selected to give a cumulative neighbourhood value of badgers captured within 500 metres of the centroid of all fragments of land (1 280 603) registered under LPIS in 2014. We assessed the relationship between CULL and the outcome using LOWESS smoothed curve [30]. Comparisons between competing transformations (linear, logarithm, quadratic, and splines) were made using Quasilikelihood Information Criteria (QIC; [31,32]). The cut-point for the spline was chosen by comparing models with increasing removal effort (CULL) in steps of 0.5 badgers per km^2^ (i.e. 0.5, 1, 1.5, 2 badgers per km^2^ etc.; [30]).

First order interaction terms were tested throughout. The interaction terms were retained if found significant using a Wald test [30].

## Results

### Historic effects of badger culling – baseline model

**Figure 1:**
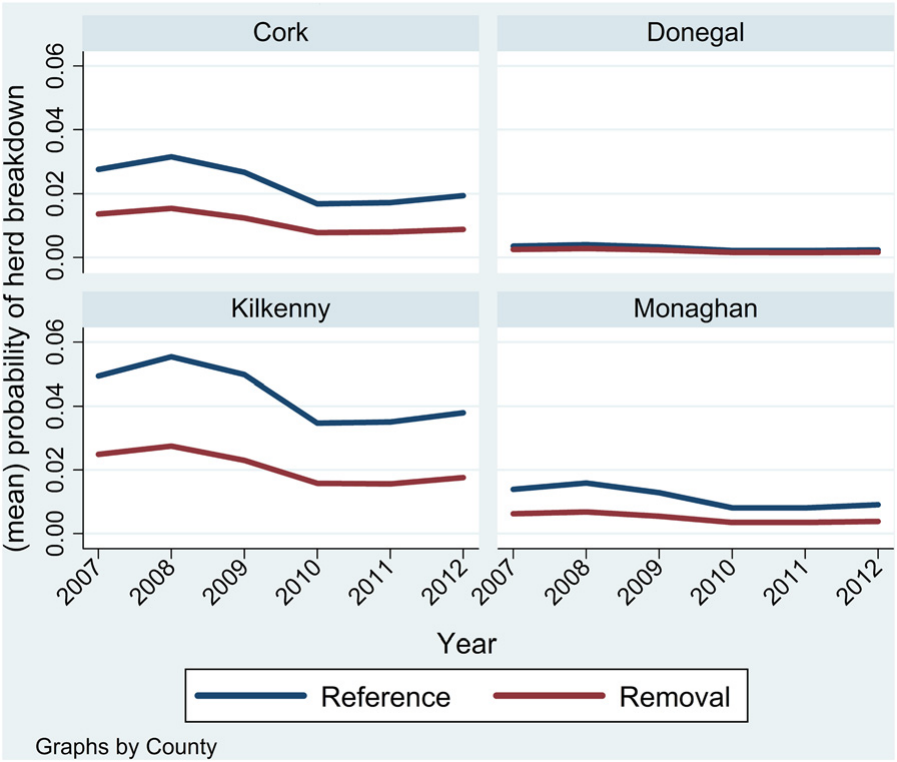
**Mean temporal trends from a model of cattle herd breakdown risk in areas within a former large-scale badger cull trial in four counties in Ireland.** Reference areas composed of farms with limited or no culling during 1997–2002; Removal areas composed of farms with intensive proactive culling during 1997–2002.

The first model we developed concentrated on herds with land within treatment areas, using an approach similar to Griffin et al. [3]. Univariable models suggested significant associations between herd breakdown risk and all predictors (*p* < 0.05). Separate univariate models for the effect of former treatment on herd breakdown risk in each county found negative associations in Kilkenny (*p* = 0.001), Monaghan (*p* = 0.001) and Cork (*p* = 0.124), but a non-significant positive association in Co. Donegal (*p* = 0.922). However, in the final multivariable model, no interaction terms were significant (*p* > 0.1). In the final model, herds within a former removal area had 0.53 the odds of having a bTB breakdown in a given year than a herd within a former reference area (*P* < 0.001; Table 3). Given the very low probability of herd breakdown in Donegal, it was difficult to detect an effect (Figure 1). Cattle herd breakdown risk fluctuated significantly (Table 3) over the study period, with an overall declining trend in risk over time (Fitted linear trend: odds ratio 0.87 per year; *p* = 0.004). Herd risk was significantly increased for herds that had a previous herd breakdown in comparison with herd that didn’t (OR: 2.250; *p* < 0.001), and with increasing herd size (log(herdsize) OR: 2.034; *p* < 0.001). Kilkenny herds had the overall greatest risk of having a breakdown, followed by Cork, Monaghan, and Donegal (Figure 1; Table 3).

**Table 3:** **Model results relating cattle herd bovine tuberculosis breakdown risk in relationship historic intensive interventions**

**Annual bTB status**	**Odds ratio**	**Std. Err.**	**z**	***P*** **> z**	**Lower 95%**	**Upper 95%**
**Reference**	1.000					
**Removal**	0.528	0.087	−3.86	0.000	0.382	0.730
**2007**	1.000					
**2008**	1.095	0.249	0.40	0.689	0.701	1.711
**2009**	0.879	0.215	−0.53	0.598	0.544	1.420
**2010**	0.579	0.159	−1.99	0.047	0.337	0.993
**2011**	0.574	0.158	−2.01	0.044	0.334	0.986
**2012**	0.611	0.165	−1.83	0.068	0.360	1.036
**Previous history**	2.250	0.485	3.76	0.000	1.474	3.434
**Log(Herdsize)**	2.034	0.202	7.16	0.000	1.675	2.471
Cork*	1.000					
Donegal*	0.388	0.175	−2.10	0.036	0.160	0.941
Kilkenny*	1.583	0.317	2.29	0.022	1.069	2.344
Monaghan*	0.682	0.148	−1.76	0.078	0.445	1.044

*Wald tests: Donegal = Kilkenny: χ^2^ (df: 1) =9.73; Prob > χ^2^ = 0.002; Donegal = Monaghan: χ^2^ (df: 1) =1.65; Prob > χ^2^ = 0.199; Kilkenny = Monaghan: χ ^2^ (df: 1) =16.88; Prob > χ^2^ < 0.001.

The effect of historic intensive proactive culls on cattle herd breakdown risk (2007–2012) in areas that were part of a former large-scale badger cull trial during 1997–2002.

### Extending the model – badger variables and non-treatment areas

#### Badger culling intensity

The overall mean badger culling intensity, as measured with the spatially explicit metric (CULL), across farm land included in this analysis was 0.59 badgers km^−2^ (SD: 1.58; IQR: 0–0.34); excluding areas within the four counties where no badgers were culled, increases the mean intensity to 1.96 badgers km^−2^ (SD: 2.37; IQR: 0.51-2.55) over the course of the study (2007–2012; Table 4). A linear regression relating culling intensity in areas with culling (log transformed) found that there was a significant difference in culling intensity between reference, removal and other areas (*p* < 0.001) and amongst counties (*p* < 0.001; and see Table 4). Overall, references areas (β = −0.12; *p* < 0.001; mean 2.04 badgers km^−2^) and removal areas (β = −0.46; *p* < 0.001; 1.14 badgers km^−2^) had lower removal intensity than other (non-treatment) areas (2.42 badgers km^−2^). Removal areas had significantly lower removal intensities than reference areas (β = −0.34; *p* < 0.001). Removal intensity varied significantly by county (*p* < 0.001); Donegal had the highest mean intensities in culling areas (2.83 badgers km^−2^), followed by Monaghan (1.94 badgers km^−2^), Cork (1.89 badgers km^−2^) and Kilkenny (1.42 badgers km^−2^). Kilkenny had reduced culling intensity in the reference area (0.72 badgers km^−2^) as the majority of the area had been part of a badger vaccine trial over the study period (see [24]). Cork had the highest culling intensity in any removal treatment area (Cork removal: 1.43 vs. mean of other removal areas: 0.95 badgers km^−2^) as a consequence of additional removal effort employed during the study period beyond targeted culling (J. O’Keeffe, pers. comm.).

**Table 4:** **Badger culling intensity metric**

		**Reference**	**Removal**	**Other**	**Total**
**County**					
**Cork**	*Mean*	2.55	1.43	1.90	1.89
	*SD*	3.03	1.38	2.21	2.21
**Donegal**	*Mean*	1.20	1.00	2.88	2.83
	*SD*	1.34	1.06	3.50	3.47
**Kilkenny**	*Mean*	0.72	0.88	1.50	1.42
	*SD*	0.80	0.94	1.74	1.68
**Monaghan**	*Mean*	1.88	0.99	2.07	1.94
	*SD*	1.88	1.09	2.19	2.08
**Total**	*Mean*	1.76	1.14	2.01	1.96
	*SD*	2.04	1.21	2.42	2.37

Culling intensity on farms in areas with removals across four counties during 2007–2012 in Ireland (these data exclude land with no culling). Reference areas were part of a former cull trial and had low historic culling; Removal areas were part of a former cull trial and had high historic culling; other areas were outside of the culling trials. Units: badgers culled km^−2^.

### *Potential density*

There was significant variation in potential density (PD) depending on former treatment type and county (Table 5). The former treatment areas within Co. Cork had a lower PD than other areas of Cork (*p* < 0.001), but were not significantly different between reference or removal areas (*p* = 0.080). In Donegal, there was no significant difference between reference, removal and other areas (*p* > 0.06). In Kilkenny, there was higher PD in the removal area relative to either reference or other areas (*p* < 0.001), but there was no difference between reference and other areas (p = 0.791). Monaghan had higher PD in reference or other areas relative to the removal area (*p* < 0.001), but there was no difference between reference and other areas (*p* = 0.538). Overall, Monaghan had the highest mean PD (0.48), followed by Kilkenny (0.45), Cork (0.44) and Donegal (0.30). A univariable model of herd breakdown risk suggested that an increase in PD was associated with increased bTB herd risk (OR: 4.25; *P* < 0.001).

**Table 5:** **Badger potential density (PD) metric**

		**Reference**	**Removal**	**Other**	**Total**
**County**					
**Cork**	*Mean*	0.37	0.39	0.45	0.44
	*SD*	0.10	0.08	0.11	0.11
**Donegal**	*Mean*	0.29	0.29	0.30	0.30
	*SD*	0.09	0.08	0.11	0.11
**Kilkenny**	*Mean*	0.44	0.49	0.44	0.45
	*SD*	0.07	0.09	0.10	0.10
**Monaghan**	*Mean*	0.49	0.42	0.50	0.48
	*SD*	0.07	0.10	0.08	0.09
**Total**	*Mean*	0.42	0.40	0.42	0.42
	*SD*	0.11	0.11	0.12	0.12

Variation in a metric of badger social group density (probability of occurrence of social groups; [19]) across farms in four counties in Ireland. Values closer to 1 represent high density areas; values closer to 0 represent low density areas. Reference areas were part of a former cull trial and had low historic culling; Removal areas were part of a former cull trial and had high historic culling; other areas were outside of the culling trials.

### *Multivariable model*

The final multivariable model with the lowest QIC value included base variables in the first model, and interaction terms with badger variables (PD and CULL; Table 6). The relationship between cattle herd risk and culling intensity was non-linear. Additional file 2 shows this relationship using a univariable smoothed regression technique (LOWESS; simply used to assess the functional form). There is an initial increase in risk with badgers removed, followed by a decline. A comparison of models using QIC suggested most support towards modelling this relationship was using a spline at a cut-point (knot) at 0.5 badgers km^−2^.

**Table 6:** **Model relating cattle herd bovine tuberculosis risk to historic interventions, metrics of badger culling and density**

**Annual bTB status**	**Odds ratio**	**Std. Err.**	**z**	***P*** **> |z|**	**Lower 95%**	**Upper 95%**
**Reference**	1.000					
**Removal**	0.527	0.086	−3.930	0.000	0.383	0.725
**Non-treatment**	0.997	0.103	−0.030	0.976	0.814	1.220
**2007**	1.000					
**2008**	0.831	0.052	−2.970	0.003	0.735	0.939
**2009**	0.567	0.039	−8.240	0.000	0.495	0.649
**2010**	0.515	0.036	−9.370	0.000	0.448	0.591
**2011**	0.582	0.040	−7.940	0.000	0.510	0.666
**2012**	0.554	0.038	−8.620	0.000	0.484	0.633
Interaction terms for year*potential density^						
2007*dens.	2.065	0.878	1.700	0.088	0.897	4.753
2008*dens.	2.672	1.582	1.660	0.097	0.837	8.526
2009*dens.	0.633	0.417	−0.700	0.487	0.174	2.300
2010*dens.	1.075	0.727	0.110	0.915	0.286	4.044
2011*dens.	0.210	0.136	−2.420	0.016	0.059	0.745
2012*dens.	0.145	0.094	−2.980	0.003	0.041	0.516
**Previous history**	2.404	0.118	17.920	0.000	2.184	2.646
**Log(Herdsize)**	1.916	0.048	26.170	0.000	1.825	2.011
**Cull(spline1)**	3.220	0.336	11.220	0.000	2.625	3.950
Interaction terms for County*culling intensity >0.5~						
CK*spline2	0.979	0.019	−1.110	0.267	0.942	1.017
DN*spline2	1.088	0.030	3.100	0.002	1.032	1.148
KK*spline2	0.839	0.053	−2.760	0.006	0.740	0.950
MN*spline2	0.997	0.049	−0.060	0.951	0.905	1.099
**Cork (CK)**	1.000					
**Donegal (DN)**	0.798	0.065	−2.780	0.005	0.681	0.935
**Kilkenny (KK)**	1.165	0.071	2.500	0.012	1.033	1.313
**Monaghan (MN)**	0.740	0.063	−3.550	0.000	0.627	0.874

**^**Overall interaction: χ^2^ (df: 5) =26.21; Prob > χ^2^ < 0.001; **~**Overall interaction: χ^2^ (df: 3) =20.77; Prob > χ^2^ < 0.001.

Final multivariable model relating factors that influence cattle herd breakdowns in four counties in Ireland in 2007–2012.

**Figure 2:**
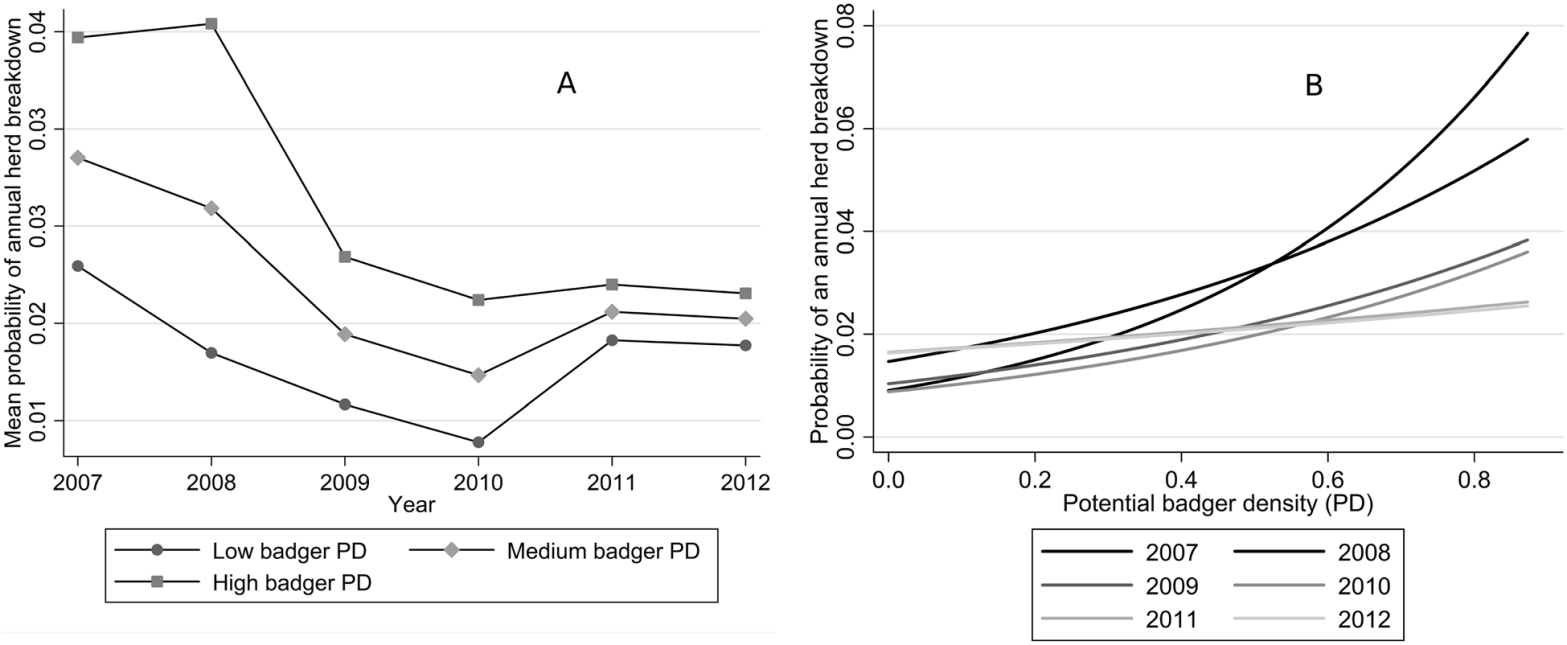
**The relationship between badger potential density (PD), as measured in social group abundance, and risk of cattle herd breakdown.** The model predicted relationship was influenced by year, with a stronger positive relationship early in the study period. **A**. The herd risk over time in farms with low, medium and high badger PD. **B**. The risk of breakdown in relation to PD for each year of the study.

**Figure 3:**
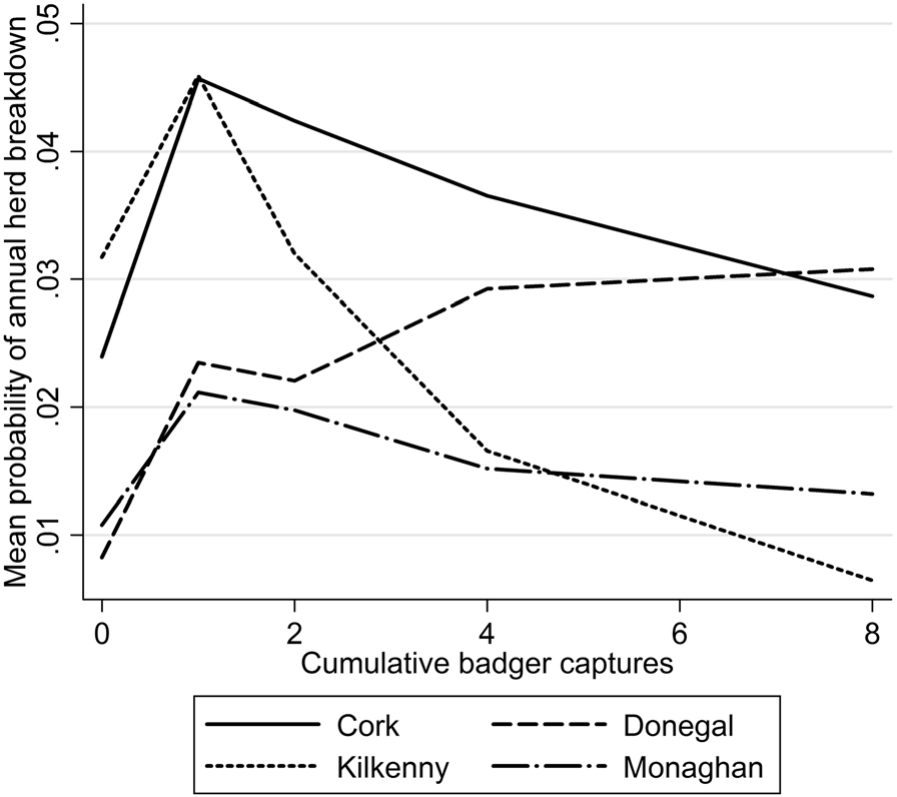
**The relationship between cattle herds breakdown risk and badger culling intensity, modelled using splines.** Herds exposed to culling are generally higher risk herds relative to herd away from culled areas. Risk declines with increased culling intensity in Counties Cork, Kilkenny and Monaghan, whereas risk increases with culling intensity in Co. Donegal.

Cattle herd breakdown risk was lower for farms in previous removal areas relative to reference areas (OR: 0.53; *P* < 0.001) and relative to non-treatment farms (OR: 0.53; *P* < 0.001). There was no significant difference in risk between farms in reference and non-treatment areas (OR: 1.00; *P* = 0.976). A history of bTB breakdowns (OR: 2.40; *P* < 0.001) and increasing herd size (OR: 1.92; *P* < 0.001) increased the risk of herd breakdown. Overall, there was a significant declining trend in herd risk across the study population during the study period (fitted linear trend: odds ratio 0.89 per year; *p* < 0.001). However, the effect of PD on herd risk varied significantly depending on year (PD*YEAR: *P* < 0.001). Early in the study there was an increased risk of bTB breakdown with increasing PD (Figure 2A); similarly, the reduction in risk over time was mainly gained from reducing the risk in high badger density areas (Figure 2B). The effect of targeted badger culling on herd breakdown risk had a curvilinear form (see Additional file 2), with significant increased risk of breakdown risk for herds exposed to culling relative to herds without culling (CULL spline 1: OR: 3.220). There was a significant interaction between the second spline of CULL and county; resulting in decreased risk in Monaghan, Kilkenny and Cork but an increased risk in Donegal (overall CULL spline 2*COUNTY: *P* < 0.001; Table 6; Figure 3).

## Discussion

During this study we found a significant overall difference in terms of cattle herd bTB risk in former removal areas relative to reference areas a decade after (2007–2012) the period of intervention (1997–2002). Univariable models suggested that this observed difference varied by county, with no detectable difference found in Co. Donegal. However, an interaction term for a dependence of treatment (removal or reference) by county was not significant in our final multivarible model. The lack of discernible difference in Co. Donegal is likely to relate to the very low probability of herds breaking down for bTB in either treatment areas (annual probability < 0.01; Figure 1). We posit that the overall observed difference in risk is a result of the historic intensive culling intervention. Long-term effects of intensive badger removal have been observed previously after interventions (of varying types and intensity) in Ireland and Britain ([17,18,32-34]). Evidence from a replicated, randomised trial in Britain found 25.7% lower (95% CI: 18.7% to 32.2% lower) incidence of confirmed breakdowns in areas that underwent intensive culling relative to non-culled areas 4.6 years (August 2011) after the end of culling operations [35]. A non-replicated project (Dorset, England) found bTB infection cleared in cattle for 7 years after intensive badger removal (trapping and gassing; [33,36]). In a second non-replicated study (Thornbury, England) a systematic removal of badgers (by gassing) in one area over 6 years resulted in a significant decline in cattle herd breakdowns, followed by a period of 11 years (1980–1991) without a confirmed breakdown [17,36]. There is an alternative possible reasoning for the observed sustained difference between the former removal areas and other areas – the positive psychological (morale) effect of being within a successful large-scale program. Farmer behaviour and farming practices may have changed as a result of being within a study (a “Hawthorne effect”), but clearly any such effect would only relate to those within removal areas where breakdown risk diminished during the initial trial (1997–2002). This could be a case of positive reinforcement feedback, where positive morale encouraged farmers to engage in lower risk activities (cattle buying behaviour) or increased disease mitigating behaviours (increased biosecurity). There is no evidence available to support such a “Hawthorne effect”, and so these potential explanations remain speculative.

We found that herd size and previous history to be significant factors relating to cattle herd breakdowns. These risk factors have been found repeatedly in previous research (e.g. [3,37] and see Skuce et al. [38] for review). Larger herds often have a larger geographic footprint, which may expose them to greater environmental risk factors (e.g. wildlife reservoir) and will also expose them to more neighbours (the risk of contiguous spread). Larger herds may also be more intensively managed and may buy-in more cattle [38]. There is also increasing risk of breakdown recurrence in larger herds, which may relate to failing to clear infection through test and slaughter [39]. We purposely used a simple metric for a history of bTB infection (similar to [3]) as we only wanted to control for this known risk factor. However, we acknowledge that the relationship between current risk and previous history may be complex, and highlight other more in-depth analysis of this issue [37,40,41].

This study represents the first study in Ireland to assess the relationship between variations in badger populations, based on a risk map and estimates of culling effort, and risk to cattle herd breakdowns. There was significant variation in our “badger” variables across treatments and locations. In general, there was less culling undertaken in former removal areas than in other treatment areas. This concurs with previous work that found lower relative abundance of badgers in removal areas relative to non-removal areas of Co. Monaghan [14]. This indicates that badger density in these areas were suppressed greatly during the four area trial (suggested to be > 85%; [16]), and the targeted culling undertaken in recent years is maintaining the badger population below carrying capacity. Badger social group density (PD) varied across counties, with Donegal being lower than the other three counties. This is most likely due to Co. Donegal being the most exposed county, with poor soils and mountainous areas [19]. There have been previous attempts in Britain to use badger abundance metrics to produce risk maps for bTB to cattle herds at national levels [28,42]. Bessell et al. [29] used one of these risk maps, the probability of main-sett presence, to model its relationship with cattle herd breakdown risk at a national scale. There was a significant increased risk of cattle herd breakdown in high-risk areas with increasing probability of badger main-sett presence. Similarly, during the present study, we found overall increased risk of herd breakdown (OR: 4.25; *P* < 0.001) with increasing badger PD. However, in the final multivariable model, there was significant variation in this effect depending on year (significant PD*YEAR interaction). Over time, there was a waning of the effect of PD on herd risk (Figure 3A), so by 2012 there was no relationship between PD and breakdown risk. This may suggest that PD (based on main sett occurrence) becomes a less robust metric of badger abundance with time. Alternatively, later in the study there may not have been enough breakdown incidents to measure an effect (as we suggest is the case specifically in Co. Donegal). A greater temporal window (for example, looking at breakdown history over two year periods) may have helped with this problem; however, we feel that such an approach would be limited in the present study due to the relatively short time series available (2007–2012).

The interaction term PD*YEAR also suggested that the general decline in risk over time depended on whether a farm was in relatively higher or lower badger density areas (Figure 3B). The greatest declines in risk occurred over time in the highest (PD) badger density areas. Other recent work has found significant declines in bTB prevalence in badgers removed from culled areas over the study period (2007–2012; [43]; Byrne et al. unpublished). Also, in Ireland, bTB levels in badgers are higher (37-50% prevalence in badgers) in areas with greater risk of cattle herd bTB breakdown, in comparison with areas with very low risk of cattle herd bTB breakdowns (15% prevalence in badgers; [44]). While there is uncertainty and limitations to our retrospective study, we propose that removals in targeted areas with higher badger density (as measured by PD) may have decreased the risk of breakdown by equalising the risk with areas with lower potential wildlife spillback risk. However, more detailed studies should be undertaken to test the robustness of these inferences.

When we included our metric of badger targeted culling into our multivariable model, we found a curvilinear relationship between culling effort and risk (see Additional file 2). There was increased risk of bTB breakdown in areas with culling (higher risk areas) relative to areas without culling (lower risk areas). This finding was expected as badgers are culled in a targeted fashion in response to cattle herd breakdowns [2,13]. Present policy dictates that badgers are only removed from farms with herd breakdowns with more than two standard bTB reactors (cattle in Ireland are tested annually using the SICTT [25]). Therefore, badgers are only removed in response to more serious breakdown episodes. In Cork, Monaghan and Kilkenny, continued culling effort was associated with a gradual declining trend in risk (post-hoc Wald test: χ^2^ (df: 3) =10.57; Prob > χ^2^ = 0.014), however, there was a trend towards increased risk within greater culling effort in Donegal (Figure 2). It is difficult to interpret the result in Donegal; however considering how few breakdowns there were in general in Donegal, there was probably little data to inform the trend. More detailed work would be needed to elucidate the effects of culling in low badger density, low cattle-herd risk areas.

### Conclusion

In this paper, we have found that there are differences in the risk profile of cattle herds located within former removal areas, relative to those without previous intensive removal, in a large-scale badger culling programme from four counties in Ireland a decade after its cessation. We found that over the period of study, there was a decline in herd breakdown risk. The greatest declines were found in areas with the highest potential badger density. Badger potential density was associated with increased herd breakdown risk; however, this relationship was only significant earlier in the study period. Targeted culling was focused on herds with increased risk relative to other herds. In three counties there were trends towards decreasing risk with increasing culling effort on targeted farms; however, this trend was reversed in Co. Donegal. Reducing risk of herd breakdowns through badger culling, while potentially effective [3,45], is recognised as not being a long-term solution to managing disease risk from wildlife hosts [2,13]. However, mixed approaches including vaccination and biosecurity strategies may help to effectively manage risk. Recent work ([43]; Byrne et al. unpublished) has found that bTB levels in badgers have decreased in Ireland after repeated widespread culling, with current badger bTB prevalence being relatively low in culled areas. This low prevalence epidemiological situation may increase the likelihood of a vaccination programme, which can act only on the number of non-infected susceptible animals in a population, to be an effective control option for managing spillback infection risk [46].

## Additional files

Additional file 1:
**Map of “four area” study locations.** Map of Ireland depicting the location of removal and reference areas of the “four area” project (1997–2012). These geographic areas were used to select herds during 2007–2012 that were exposed to a former treatment during the “four area” project. Additional file 2:
**LOWESS (locally weighted regression) plot.** LOWESS (locally weighted regression; bandwidth 0.8; 20% random sample of all data) plot for the relationship between herd breakdown and cumulative number of badgers captured around cattle herds in four counties in Ireland in 2007–2012. A. Using all data up to 15 badgers km^−2^. B. Using data up to 10 badgers km^−2^.
