# Supplementary figures and images for: Risk of tuberculosis cattle herd breakdowns in Ireland: effects of badger culling effort, density and historic large-scale interventions
Source: Vet Res. 2014 Oct 26;45(1):109. doi: 10.1186/s13567-014-0109-4 (PMC4230509; doi:10.1186/s13567-014-0109-4)

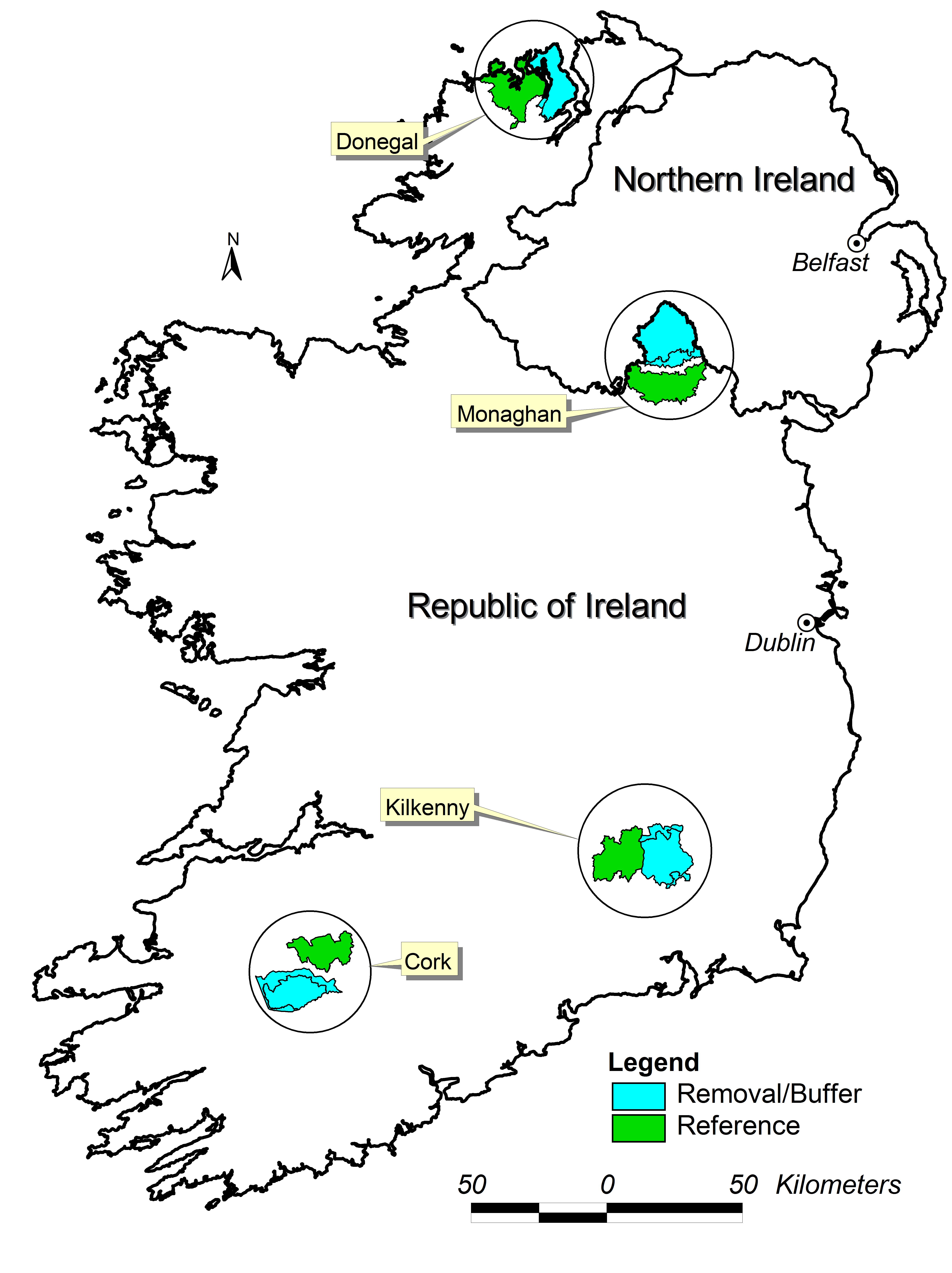

Supplement: Additional file 1: — Map of “four area” study locations. Map of Ireland depicting the location of removal and reference areas of the “four area” project (1997–2012). These geographic areas were used to select herds during 2007–2012 that were exposed to a former treatment during the “four area” project. [file 13567_2014_109_MOESM1_ESM.jpeg]

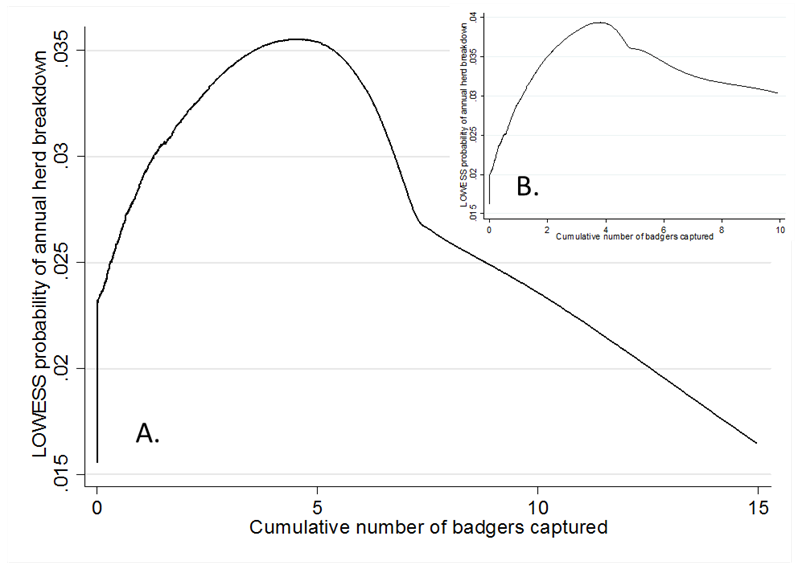

Supplement: Additional file 2: — LOWESS (locally weighted regression) plot. LOWESS (locally weighted regression; bandwidth 0.8; 20% random sample of all data) plot for the relationship between herd breakdown and cumulative number of badgers captured around cattle herds in four counties in Ireland in 2007–2012. A. Using all data up to 15 badgers km−2. B. Using data up to 10 badgers km−2. [file 13567_2014_109_MOESM2_ESM.tiff]
